# Legume–grass mixtures improve biological nitrogen fixation and nitrogen transfer by promoting nodulation and altering root conformation in different ecological regions of the Qinghai–Tibet Plateau

**DOI:** 10.3389/fpls.2024.1375166

**Published:** 2024-06-13

**Authors:** Feng Luo, Wenbo Mi, Wenhui Liu

**Affiliations:** ^1^Key Laboratory of Superior Forage Germplasm in the Qinghai-Tibetan Plateau, Qinghai Academy of Animal Husbandry and Veterinary Sciences, Qinghai University, Xining, China; ^2^Laboratory of Tibetan Plateau Germplasm Resources Research and Utilization, College of Agricultural and Forestry Sciences, Qinghai University, Xining, China

**Keywords:** cropping pattern, legume forage, symbiotic nitrogen fixation, rhizoma, root system, soil physicochemical, ecoregion

## Abstract

**Introduction:**

Biological nitrogen fixation (BNF) plays a crucial role in nitrogen utilization in agroecosystems. Functional characteristics of plants (grasses vs. legumes) affect BNF. However, little is still known about how ecological zones and cropping patterns affect legume nitrogen fixation. This study’s objective was to assess the effects of different cropping systems on aboveground dry matter, interspecific relationships, nodulation characteristics, root conformation, soil physicochemistry, BNF, and nitrogen transfer in three ecological zones and determine the main factors affecting nitrogen derived from the atmosphere (Ndfa) and nitrogen transferred (Ntransfer).

**Methods:**

The ^15^N labeling method was applied. Oats (*Avena sativa* L.), forage peas (*Pisum sativum* L.), common vetch (*Vicia sativa* L.), and fava beans (*Vicia faba* L.) were grown in monocultures and mixtures (YS: oats and forage peas; YJ: oats and common vetch; YC: oats and fava beans) in three ecological regions (HZ: Huangshui Valley; GN: Sanjiangyuan District; MY: Qilian Mountains Basin) in a split-plot design.

**Results:**

The results showed that mixing significantly promoted legume nodulation, optimized the configuration of the root system, increased aboveground dry matter, and enhanced nitrogen fixation in different ecological regions. The percentage of nitrogen derived from the atmosphere (%Ndfa) and percentage of nitrogen transferred (%Ntransfer) of legumes grown with different legume types and in different ecological zones were significantly different, but mixed cropping significantly increased the %Ndfa of the legumes. Factors affecting Ndfa included the cropping pattern, the ecological zone (R), the root nodule number, pH, ammonium-nitrogen, nitrate-nitrogen, microbial nitrogen mass (MBN), plant nitrogen content (N%), and aboveground dry biomass. Factors affecting Ntransfer included R, temperature, altitude, root surface area, nitrogen-fixing enzyme activity, organic matter, total soil nitrogen, MBN, and N%.

**Discussion:**

We concluded that mixed cropping is beneficial for BNF and that mixed cropping of legumes is a sustainable and effective forage management practice on the Tibetan Plateau.

## 1 Introduction

Soil nitrogen is a key factor influencing crop growth in cropping systems (Mcgraw et al., 2008; Thilakarathna et al., 2012). Modern agriculture achieves high yields by using large amounts of inorganic nitrogen fertilizer and non-renewable resources, a practice that is now being questioned (Hatano et al., 2002). Studies have shown that such production practices negatively affect the nitrogen cycle and nitrogen balance (Moorhead et al., 2013), are costly in terms of public health and environmental safety (Tilman et al., 2002; Francis et al., 2016), and are a serious impediment to sustainable agricultural development (Song et al., 2020). Thus, agroecology emphasizes the design of cropping systems using ecosystem services and the sustainability of agricultural production systems (Clergue et al., 2005; Faucon et al., 2017; Olounlade et al., 2017). Biological nitrogen fixation (BNF) by legumes is an important way to replenish soil nitrogen (Yao et al., 2019). Mixed grass and legume forage cropping systems, which significantly optimize the cropping system by increasing plant diversity and improving soil health (Crème et al., 2015; Zhao et al., 2015), are a way to develop sustainable ecological agriculture (Luo et al., 2023).

Legume forage plays a crucial role in livestock development by providing a protein-rich source for grass-fed livestock and by improving soil quality by symbiotic nitrogen fixation with soil rhizobacteria (Rochon et al., 2010; Rispail et al., 2015). Including legumes in mixed cropping systems increases crop yield (Tilman et al., 2002), improves forage quality (Tahir et al., 2023), increases resource utilization (Loreau et al., 2001) and soil quality (Wichern et al., 2007; Hinsinger et al., 2009), and maintains the nitrogen balance in the soil system (Ledgard and Steele, 1992), which reduce chemical inputs and environmental pollution (Hinsinger et al., 2009; Frankow-Lindberg and Dahlin, 2013). This is because prokaryotic microorganisms are catalyzed by nitrogen-fixing enzymes in mixed cropping systems of grasses and legumes, which reduces atmospheric nitrogen to plant-available nitrogen (ammonia), providing an additional source of nitrogen for the grasses (Fitter, 1994). At the same time, nitrogen is transferred from high-nitrogen-producing plants (Leguminosae) to low-nitrogen-producing plants (Gramineae) due to the reservoir source relationship, and this mechanism of nitrogen transfer frees Gramineae from nitrogen limitations (Jalonen et al., 2009; Poffenbarger et al., 2015). In addition, due to interspecific competition among crops, competition for soil nitrogen from grass crops stimulates legumes to fix more nitrogen from the atmosphere for crop growth and development and also reduces the nitrogen deterrent effect of legumes (Mtambanengwe and Mapfumo, 1999; Peoples et al., 2015).

Plants use interspecific complementarity and interspecific competition to access soil resources and promote rhizomatous nitrogen fixation through positive plant–root–soil interactions (Duchene et al., 2017). Grass–bean mixed grasslands rely on the symbiotic relationship between rhizomes attached to legume root systems and soil nitrogen-fixing bacteria to fix nitrogen, which affects the soil carbon/nitrogen balance and improves nitrogen utilization and mineralization rates (Odu and Akerele, 1973; Sainju et al., 2003). In addition, mixes of grassland increase organic matter input and beneficial soil microorganisms to maintain the soil nutrient balance (Fornara and Tilman, 2008; Fornara et al., 2009; Deyn et al., 2011). However, promoting and suppressing nitrogen fixation efficiency depends on the pattern of competition for soil nitrogen between the root systems of grasses and legumes, which is a dynamic equilibrium (Haynes, 1980; Stern, 1993; Bouma et al., 2001). In addition, the efficiency of nitrogen transfer from legumes to grasses is related to root system secretions and the characteristics of the rhizomes. A high rate of BNF does not represent a high nitrogen transfer efficiency (Rui et al., 2022). BNF in legume crops is affected by biological factors (crop type and inter-root mycorrhizal flora) (Meng et al., 2015) and environmental factors (moisture, temperature, and soil nutrients) (Dollete et al., 2023). Therefore, it is necessary to analyze the nodulation characteristics, root phenotypic traits, soil physicochemical properties, and interspecific relationships of grasses planted in a mixed cropping system of grasses with different species of legumes in different ecological zones to assess BNF and nitrogen translocation capacity of leguminous pasture grasses and the productivity of grasses to improve livestock production.

The Tibetan Plateau, with an average elevation of 4,000 m above sea level (Li et al., 2022), is known as the “third pole”. The unique geographical location and climatic conditions of the Tibetan Plateau create an alpine meadow ecosystem (Zhang et al., 2014). The alpine meadows of the Tibetan Plateau are the largest alpine grassland distribution area in the world, covering an area of approximately 2.27 × 10^6^ km^2^ (Liu et al., 2023), which serves as an ecological barrier and an important source of forage (Hu et al., 2021). Livestock husbandry is the leading industry in this area (Wei et al., 2017). In recent years, human activities and climatic factors have led to grassland degradation, and bare vegetation has reduced the total amount of nitrogen fixation in natural grasslands and lowered the ecological service function (Harris, 2010; Ren et al., 2014; Xue et al., 2017). Therefore, optimizing cropping systems with ecologically sound forage is essential to restore the grasslands and develop animal husbandry practices (Dong et al., 2010). Introducing a mixed forage cropping system with legumes improves forage quality and replaces inorganic nitrogen fertilizer inputs (Tahir et al., 2022). Symbiotic nitrogen fixation accounts for 70% of the overall BNF in agroecosystems (Herridge et al., 2008), and leguminous crops provide 32–149 kg·hm^−2^ of nitrogen to growing crops through BNF, increasing the total amount of nitrogen in the nitrogen cycle of the agroecosystem (Mueller and Thorup-Kristensen, 2001). However, different cropping patterns and crop types have different effects on the rates of BNF and nitrogen transfer in leguminous crops. Climate is another key factor influencing BNF in leguminous crop fields (Liu et al., 2019). The altitude and climate of the different alpine ecological zones are different, and microclimates predominate. However, the forage production capacity, BNF, and nitrogen transfer rates of different species of leguminous forage mixed with oats in different ecological zones are unclear. Therefore, there is a need to study BNF and nitrogen transfer in legumes under various cropping patterns and in different ecological zones to increase forage production and to mitigate the negative impacts on the environment.

This study investigated the effects of different cropping patterns on aboveground dry matter, interspecific relationships, nodulation characteristics, root phenotypic traits, soil physicochemical properties, BNF, and nitrogen transfer in different alpine ecological zones. The results of this study will guide mixed forage cropping in the Qinghai region of China, which will reduce hazards to the environment and promote sustainable development of agroecosystems.

## 2 Materials and methods

### 2.1 Study site

**Figure 1:**
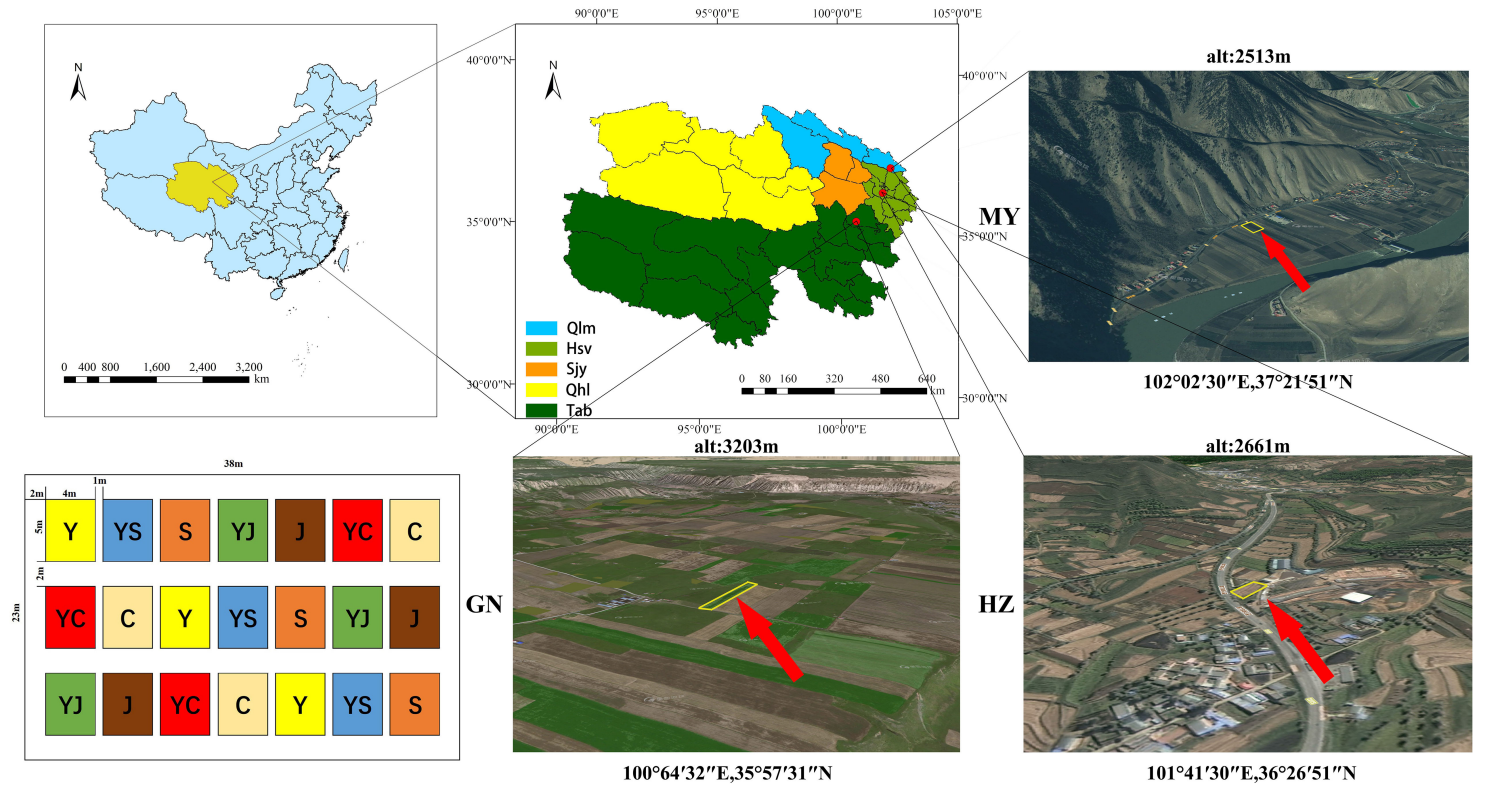
Study area and cropping map.

The experiment was conducted at a planting site in each of the three ecological zones in Qinghai Province, China (**Figure 1**):

1) HZ (Huangzhong County in Huangshui Valley): located in Garur Village, Garur Township, Tumen Pass Township. No irrigation was present, and it was a typical shallow mountain cultivation area. The area has a highland continental climate, with a short warm season and a long winter. The average annual temperature and precipitation are 5.3°C and 490 mm, respectively, and the soil type is calcium chestnut soil, with oats and legume forage as the previous crops in 2022.

2) GN (Guinan County, Sanjiangyuan District): located in Tashiu Village, Tashiu Township. No irrigation was present; it has a highland continental climate with long winters and short summers, and a cold and humid climate. The average annual temperature and precipitation are 3.2°C and 403 mm, respectively, and the soil type is clay loam, with oats and legume pasture as the previous crops in 2022.

3) MY (Menyuan County, Qilian Mountain Basin): located in Xianmi Township, Xianmi Township. No irrigation was present, and the plateau continental climate is typical of the cold, warm, and humid climate of the plateau. It has a snowy and windy spring, a cool and rainy summer, a mild and short autumn, and a cold and long winter. The average annual temperature and precipitation are 4.2°C and 518 mm, respectively, and the soil type is black calcium soil, with oats and legume pasture as the previous crops in 2022. The soil physicochemical properties are shown in **Table 1**.

**Table 1:** Soil physicochemical properties before sowing in the study area.

Soil indicators	Ecological region basic information		
	HZ	GN	MY
SOM (g·kg^−1^)	34.4	34.47	50.1
TN(g·kg^−1^)	2.2	2.4	3.1
TP(g·kg^−1^)	2.5	1.7	2
TK (g·kg^−1^)	24.4	18.1	21.1
AN(mg·kg^−1^)	120	105	124
APs(mg·kg^−1^)	27.6	20.3	26.1
AK (mg·kg^−1^)	290	244	258

### 2.2 Experimental design

The trial employed a randomized block group design, comprised of seven treatments. Each treatment had three replication plots, for a total of 21 plots. The plot was the experimental unit with an area of 20 m^2^ (5 m × 4 m). The seeds were provided by the Qinghai Academy of Animal Husbandry and Veterinary Science (**Table 2**).

**Table 2:** Planting systems and sowing rates.

Treatments	Crop and species	Seeding quantity/g·m^−2^
Y	Oat (Qinghai 444)	22.50
S	Forage peas (Qingjian No. 1)	11.16
J	Common vetch (Ximu No. 324)	12.00
C	Fava bean (Qingcan No. 22)	16.53
Y/S	Oat/Forage peas	13.50/4.45
Y/J	Oat/Common vetch	15.8/6.61
Y/C	Oat/Fava bean	13.50/6.61

The HZ, MY, and GN test sites were sown based on the local climate and sowing dates. A total of 75 kg·ha^−1^ of urea (46% N) and 150 kg·ha^−1^ of calcium superphosphate (12% P_2_O_5_) were applied as basal fertilizers before sowing. The sowing amounts are listed in **Table 1** (Xiang, 2022). Field management practices were consistent with other crops. Weeds were controlled twice manually.

To assess nitrogen fixation by the plants in the mixed sowing plots, the plants were ^15^N-marked 2 weeks before harvest using the following method. A 0.25-m^2^ marking strip of uniform length was selected for each single and mixed sowing plot. A perforated plastic sheet was used to identify the marking holes in the area using pieces of wire 15 cm apart, for a total of 28 marking holes. Then, a syringe with a 3-cm needle was attached, and 2 ml of a 0.08 g/m double-labeled ^15^NH_4_^15^NO_3_ (99%, supplied by the Shanghai Institute of Isotope Chemistry) solution was aspirated into the labeled holes for ^15^N labeling. After completion, four thick wires of 50 cm length were used to fix the position of the ^15^N markers for later sampling.

### 2.3 Sampling and measurements

Sampling was conducted at the oat milky stage, when the quality of the oat forage was optimum. Due to the different climates and altitudes at the three sites, the growing and harvesting periods of the crops were different. The HZ, MY, and GN sites were harvested, and soil samples were collected on 21 August, 28 August, and 9 September 2023, respectively.

#### 2.3.1 Sampling

Whole plot yield measurements were used. The plants were mowed to the ground and weighed fresh (for mixed crops, the two crops were weighed separately). A 1,000 g sample of fresh forage was collected from each plot and brought back to the laboratory in air-dried bags, air-dried at 105°C for 30 min, then air-dried at 65°C to constant weight. The samples were ground in a ball mill to determine ^15^N abundance and total nitrogen (TN) content. The soil was carefully excavated with a spade to a depth of 35 cm along the root system, and the large pieces of soil were shaken off. Five intact legume and oat root systems were selected from each plot, the number of root tumors were counted, and the fresh weight of the root tumors were weighed. The root tumors and root systems were also brought to the laboratory for determination of nitrogen-fixing enzyme activity and scan of root structure. Then, the roots were dried at 105°C for 30 min and baked in an oven at 80°C to constant weight to determine root biomass. Soil samples were collected from the 0–10-cm layer using the five-point method. A portion of fresh soil was used to determine microbial biomass nitrogen (MBN) content, while the rest of the soil samples were dried naturally and sieved to determine other soil indicators.

#### 2.3.2 Measurements

Plant ^15^N abundance was determined by mass spectrometry (DELTAplus XP, Thermo Finnigan Electron Corp., Mannheim, Germany). Root tumor nitrogen fixing enzyme activity was determined by acetylene reduction method, and nitrogen fixing enzyme activity was expressed as acetylene concentration (U/g) (Khan et al., 1994). TN in the plants and soil was determined by the Kjeldahl method. Soil organic matter (SOM) was determined by redox titration with K_2_Cr_2_O_7_. Soil ammonium nitrogen (ANN) was determined by the indophenol blue colorimetric method. Soil nitrate–nitrogen (NN) was assessed using the phenol disulfonic acid colorimetric method. Soil MBN was determined by the chloroform fumigation leaching method (Joergensen, 1996). Soil pH was determined by potentiometry (water–soil ratio, 2.5:1). Roots were scanned using a dual-light source color scanner (Sinocrystal ScanMaker i800 plus, Hangzhou Wanshen Inspection Science and Technology Co.)

### 2.4 Data collection and analysis

The land equivalent ratio (LER) of a mixed cropping system was calculated using Equation 1:


(1) $$\text{LER}=\frac{{\text{L}}_{\text{YI}-\text{Y}}}{{\text{L}}_{\text{Y}}}+\frac{{\text{Y}}_{\text{YI}-\text{I}}}{{\text{L}}_{\text{I}}}$$

where Y represents oats and I represent legume forage. I = S, forage pea; I = J, common vetch; and I = C, fava bean. L_YI-Y_ and L_YI-I_ represent the aboveground dry matter mass of mixed-crop oats and the legume forage, respectively, and L_Y_ and L_I_ represent the aboveground dry matter mass of monocrop oats and the monocrop legume forage, respectively. When LER > 1, the aboveground dry matter mass of the mixed crop was more advantageous than that of the monoculture and *vice versa*.

Relative abundance of ^15^N (δ^15^N) was calculated using Equation 2 (Yoneyama et al., 1986):


(2) $$\delta^{15}N\left(\%\right)=\frac{Atom\%{\;}^{15}N\left(sample\right)-Atom\%{\;}^{15}N\left(standard\right)}{Atom\%{\;}^{15}N\left(standard\right)}\times 1000$$

where δ^15^N is the relative abundance of ^15^N in the sample; atom%^15^N (sample) is the atomic abundance of ^15^N in the sample; and atom% ^15^N (standard) is the atmospheric abundance of ^15^N (0.3663%), which is used as the standard isotope abundance of ^15^N.

The %N_dfa_ of the legume forage and the proportion of N transferred from the legume forage to oats (%N_transfer_) were calculated using Equation 3 and Equation 4, respectively (Herridge et al., 1995; Neumann et al., 2009):


(3) $${\text{\%N}}_{\text{dfa}}=\left(1-\frac{{\text{A\%E}}_{\text{YI}-\text{I}}}{{\text{A\%E}}_{\text{Y}}}\right)\text{ \%}$$

(4) $${\text{\%N}}_{\text{transfer}}=\left(1-\frac{{\text{A\%E}}_{\text{YI}-\text{Y}}}{{\text{A\%E}}_{\text{I}}}\right)\text{ \%}$$

where A%E_Y_ and A%E_I_ are the δ^15^N of the single-crop oat and legume forage, respectively. A% E_YI-Y_ and A% E_YI-I_ are the δ^15^N of mixed-crop oat and legume forage, respectively.

### 2.5 Statistical analysis

Differences in aboveground biomass, nitrogen yield, rhizome traits, root morphology, soil physicochemical properties, BNF, and nitrogen transfer between cropping systems and the cropping areas were tested using two-way analysis of variance followed by Duncan’s multiple comparison test using SPSS 20.0 software (SPSS Inc., Chicago, IL, USA). A *p*-value< 0.05 was considered significant. Graphs were plotted using Origin 2021 software (OriginLab, Northampton, MA, USA). Relationships between the soil’s physical properties, rhizome characteristics, climatic factors, and aboveground biomass and variables, such as BNF and nitrogen transfer, were determined by calculating Mantle’s test and Pearson’s correlation coefficients. Statistical analyses and mapping were performed using R 4.3.1 for Windows and the “ggplot2”, “linkET”, “dplyr”, and “piecewiseSEM” software packages (The R Foundation for Statistical Computing, Vienna, Austria).

## 3 Results

### 3.1 Effect of the cropping pattern on aboveground dry biomass and nitrogen accumulation in the different ecological zones

The cropping pattern, ecological zone, and their interactions had extremely significant (*p*< 0.01) or significant (*p*< 0.05) effects on aboveground dry biomass (**Table 3**). In all ecological zones, total aboveground dry biomass was significantly higher in all three mixed cropping patterns than in the oat monoculture, and the differences among the three mixed cropping combinations were significant in the same ecological zone. In addition, total aboveground dry matter in the three ecological zones was in the order of MY (770.79 g·m^−2^) > HZ (750.04 g·m^−2^) > GN (740.90 g·m^−2^), and tended to decrease with increasing altitude. The LER of mixed cropping was >1 in all ecological zones, indicating that the total aboveground biomass of the mixed cropping system was more advantageous than that of oat monoculture. The cropping pattern with the highest aboveground total dry matter in each ecological zone was in the order of HZ (YS), GN (YC), and MY (YS), which were 16.37%, 17.07%, and 12.16% higher than that of oat monoculture, respectively.

**Table 3:** Aboveground dry biomass and the land equivalent ratio (LER) of monoculture and mixed cropping in the three ecological zones.

Ecological region	Cropping system	Oats (g·m^−2^)	Leguminosae (g·m^−2^)	Total (g·m^−2^)	LER
HZ	Y	746.75 ± 8.99Ba	–	746.75 ± 8.99Bc	–
	YS	693.61 ± 5.83Bb	175.17 ± 8.78Ac	868.78 ± 14.45Ba	1.19
	S	–	658.3 ± 16.18Aab	658.3 ± 16.18Ade	–
	YJ	710.27 ± 7.14Ab	114.72 ± 11.56Abd	824.99 ± 8.48Bb	1.13
	J	–	644.08 ± 32.59Ab	644.08 ± 32.59Ae	–
	YC	674.1 ± 15.2Bc	145.19 ± 8.65Bc	819.28 ± 23.17Bb	1.11
	C	–	688.12 ± 11.55Aa	688.12 ± 11.55Ad	–
GN	Y	724.55 ± 12.25Ba	–	724.55 ± 12.25Bc	–
	YS	677.47 ± 2.53Cb	155.47 ± 6.68Bc	832.94 ± 4.19Ca	1.18
	S	–	629.68 ± 4.74Bb	629.68 ± 4.74Be	–
	YJ	693.27 ± 5.81Bb	110.48 ± 2.55Bd	803.75 ± 7.52Cb	1.13
	J	–	618.83 ± 18.3Ab	618.83 ± 18.3Ae	–
	YC	685.36 ± 20.23ABb	162.9 ± 5.98Ac	848.26 ± 24.07Aa	1.18
	C	–	686.09 ± 12.61Aa	686.09 ± 12.61Ad	–
MY	Y	799.9 ± 12.25Aa	–	799.9 ± 12.25Ac	–
	YS	726.06 ± 6.56Ab	171.03 ± 4.33Ac	897.09 ± 6.2Aa	1.16
	S	–	675.3 ± 13.97Aa	675.3 ± 13.97Ad	
	YJ	713.2 ± 7.79Abc	131.31 ± 9.63Ad	844.51 ± 3.2Ab	1.10
	J	–	630.91 ± 19.55Ab	630.91 ± 19.55Ae	
	YC	707.41 ± 3.34Ac	153.82 ± 4.3Abc	861.23 ± 5.07Ab	1.11
	C	–	686.61 ± 14.96Aa	686.61 ± 14.96Ad	–
Ecological region (R)		**	*	**	–
Cropping system (P)		**	**	**	–
R * P		**	*	**	–

Lowercase letters indicate significant differences in the same ecological region for different planting patterns, while uppercase letters represent significant differences in the same planting patterns for different ecological regions (p< 0.05). The same as below. * and ** represent significant differences at the 0.05 and 0.01 levels, respectively.

**Figure 2:**
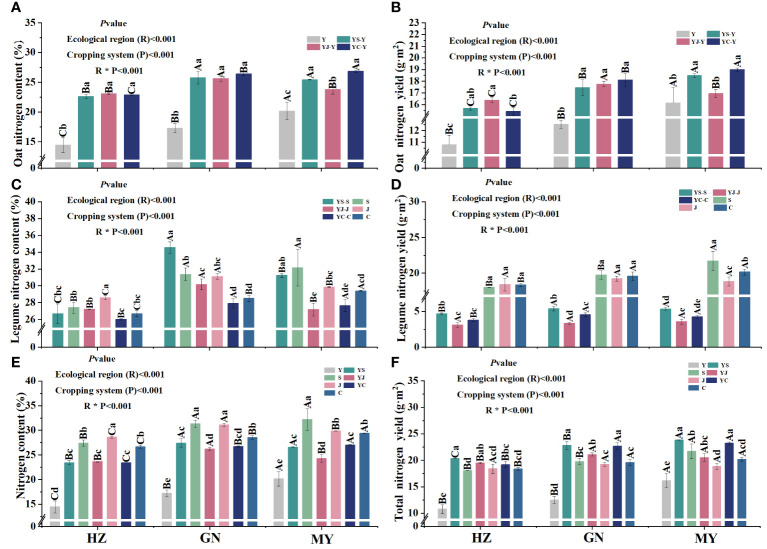
Nitrogen content and accumulation in aboveground dry matter of oats and legumes in the different ecological zones under different cropping patterns. **(A)** Nitrogen content of aboveground dry matter in oats. **(B)** Nitrogen accumulation of aboveground dry matter in oats. **(C)** Nitrogen content of aboveground dry matter in pulses. **(D)** Nitrogen accumulation of aboveground dry matter in pulses. **(E)** Nitrogen content of aboveground dry matter in cropping systems. **(F)** Nitrogen accumulation of aboveground dry matter in cropping systems.

The cropping pattern, ecological zone, and their interaction had significant (*p*< 0.05) effects on nitrogen content and nitrogen accumulation of the aboveground biomass (**Figure 2**). In all ecological zones, nitrogen content and nitrogen accumulation of oats grown in mixes were significantly higher than that of oats grown in monoculture, and the differences were significant among the ecological zones. In addition, no significant differences in nitrogen content or nitrogen accumulation of oats grown in the three mixes were detected in the same ecoregion (**Figures 2A, B**). In all ecological zones, nitrogen content and nitrogen accumulation of the mixed legume forages were significantly higher than their respective counterparts in monoculture (**Figures 2C, D**). N content and total N accumulation were greater for mixed cropping than oat monocropping (**Figures 2E, F**).

### 3.2 Effect of the ecological zone planting pattern on root tumor characteristics of legume forage grasses

**Figure 3:**
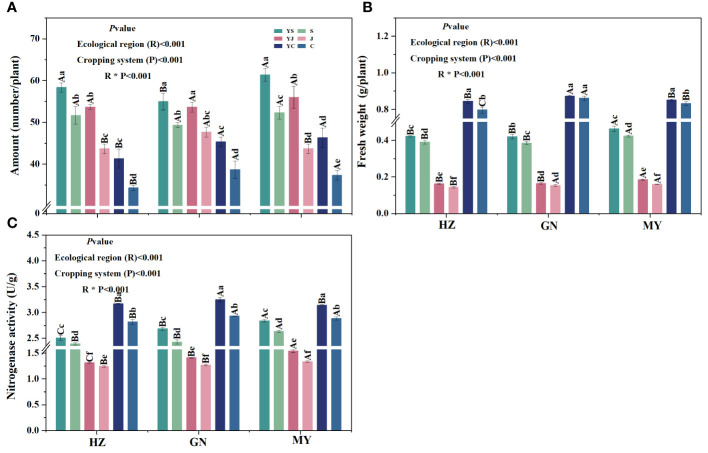
Characteristics of legume root tumors under different cropping patterns in different ecological zones. **(A)** Root tumor number. **(B)** Root tumor fresh weight. **(C)** Nitrogen-fixing enzyme activity.

The cropping pattern, ecological zone, and their interactions had significant (*p*< 0.05) effects on root tumor number, root tumor fresh weight, and nitrogen-fixing enzyme activity (**Figure 3**). In all ecological zones, the number of tumors of mixed legume forages was significantly greater than that of the corresponding monocultures, and the highest number of tumors was found in the YS cropping 7pattern, followed by YJ. The number of rhizomes on C monocultures was significantly lower than that of the other cropping patterns (**Figure 3A**). Significant differences in tumor weight and nitrogen-fixing enzyme activity were observed in all ecological zones among the three legumes, and all showed the pattern of broad bean > forage pea > arrow end pea. In addition, in the same ecoregion, the tumor weight and nitrogen-fixing enzyme activity of the legume forage grasses grown in monoculture were significantly lower than those of the corresponding mixes, except for the root tumor weights of the C and YC cropping patterns of GN (**Figures 3B, C**).

### 3.3 Effect of the ecological region planting pattern on forage root characteristics

**Figure 4:**
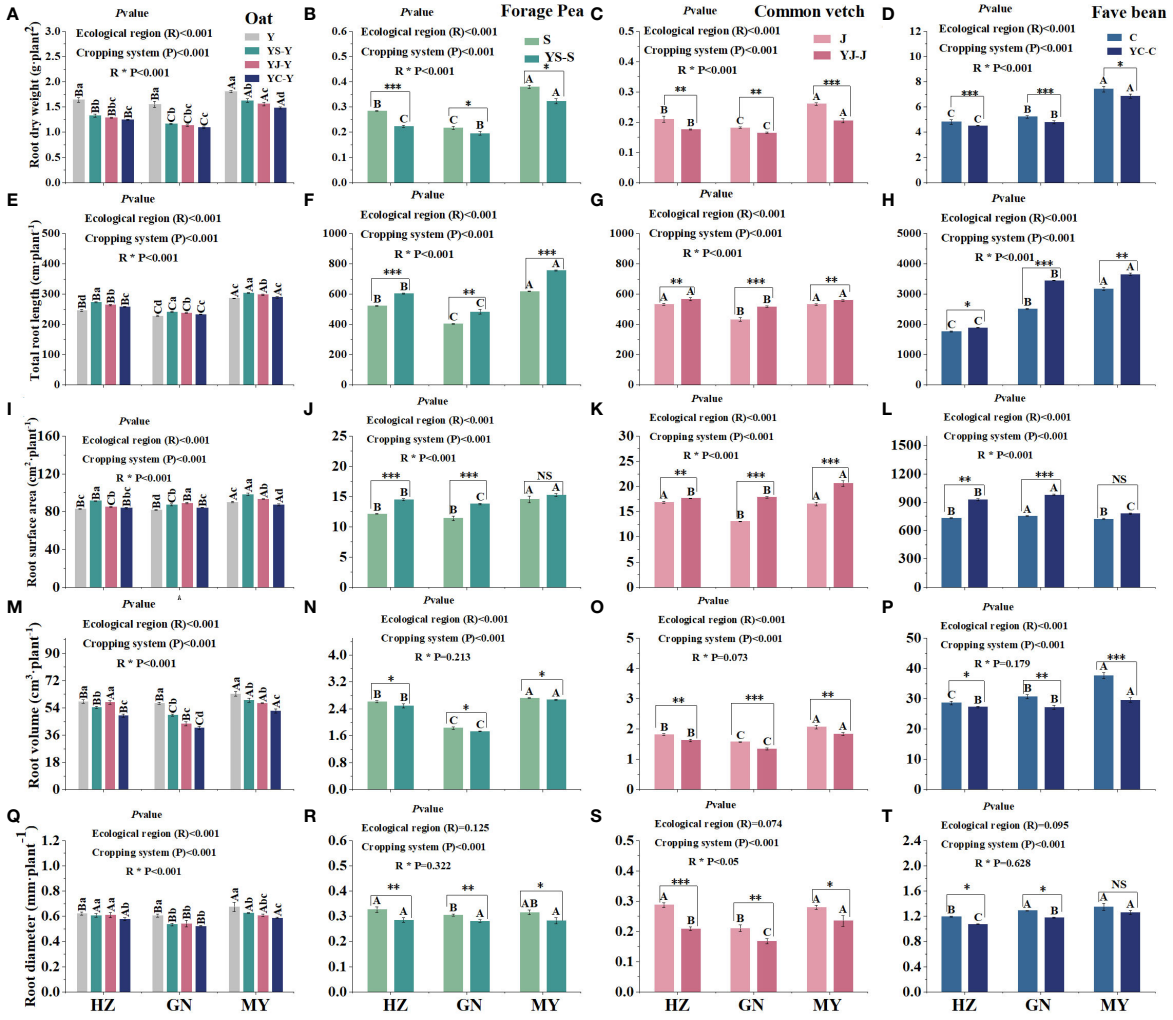
Root system characteristics of monoculture and mixed cropping of oat and legume forages in different ecological zones. (**A–D**) Root dry weight. (**E–H**) Total root length. (**I–L**) Root area. (**M–P**) Root volume **(Q–T)** and root diameter. Lowercase letters indicate significant differences in the same ecological regions for different planting patterns, whereas uppercase letters represent significant differences in the same planting patterns for different ecological regions (*p*< 0.05). * denotes a significant difference between legume monoculture and mixture in the same ecoregion, ∗ denotes 0.01< *p*< 0.05, ∗∗ denotes *p*< 0.01, and ∗∗∗ denotes *p*< 0.001.

Root dry weight and root volume were significantly higher in monoculture than those in the corresponding mixtures in all ecological zones and were highest in fava beans, followed by oats. Moreover, root dry weight and root volume were significantly higher in MY than in HZ or GN (**Figures 4A–D, M–P**). In contrast, in all ecological zones, root length and root surface area were significantly higher in the mixed cropping than in the corresponding monoculture cropping pattern. Root length and surface area revealed an overall pattern of broad bean > oat > arrow end pea > pea. In addition, root length and root surface area among regions were in the order of MY > HZ > GN (except broad bean) (**Figures 4E–I, G, K, L**). Root diameter was significantly higher in monoculture oats than in the mixed cropping pattern, and the YC cropping pattern had the smallest root diameter in oats. The root diameter of oats between regions followed the same trend as root dry weight and root volume, both indicating that MY was significantly higher than HZ and GN (**Figure 4Q**). Additionally, the root diameters of all three legume mixtures were lower than those of the corresponding monocultures, suggesting that root growth of the legumes was inhibited in the mixed cropping system (**Figures 4R–T**). In addition, the root systems of crops in different ecological zones varied considerably, e.g., the root surface area of broad bean was largest in the GN region, whereas the root surface area of arrow end pea and oat was largest in the MY region (**Figures 4I, K, L**).

The planting pattern, the ecological zone, and their interactions had significant or extremely significant (*p<* 0.05) effects on root dry weight, root length, root area, root volume, and root diameter for all crops, except for the ecological zones, which had no significant effect on the diameter of the three types of beans, or the volume of the three types of beans as a result of the interaction between the planting pattern and the ecological zone (**Figure 4**).

### 3.4 Effect of cropping pattern on soil physicochemical properties in different ecological zones

**Figure 5:**
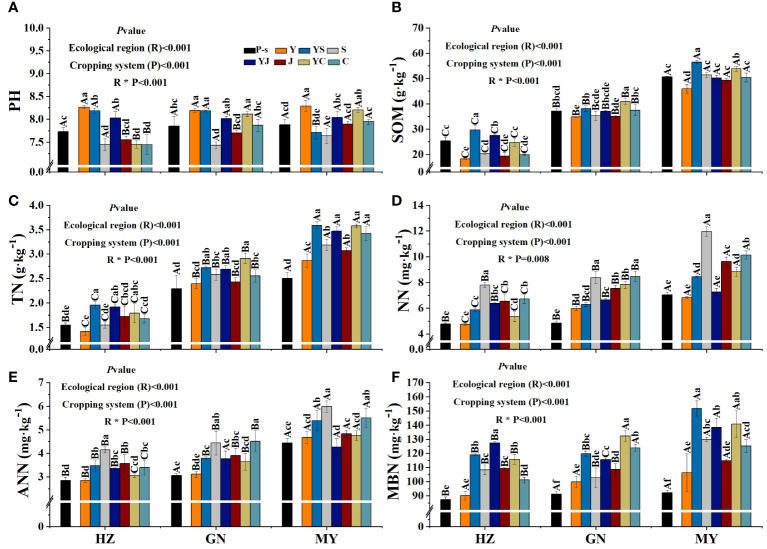
Soil physicochemical properties of the cropping patterns in different ecological regions. **(A)** Soil pH. **(B)** Soil organic matter. **(C)** Soil total nitrogen. **(D)** Soil ammonium nitrogen. **(E)** Soil nitrate nitrogen. **(F)** Soil microbial nitrogen.

The planting pattern, ecological zone, and their interaction had extremely significant (*p<* 0.01) effects on pH, SOM, TN, NN, ANN, and MBN, except for the interaction between the planting pattern and the ecological zone, which did not have a significant effect on NN (**Figure 5**).

Mixed cropping tended to reduce soil pH compared to oat monoculture, but the difference was not significant (**Figure 5A**). Monocropping and mixed cropping of the three legumes increased SOM compared to oat monocropping in all ecological zones, with legume monocropping the highest, followed by mixing. SOM of the mixed crops was significantly higher than that of oat monoculture. In addition, the cropping patterns with the highest SOM in each ecological zone were HZ (YS), GN (YC), and HZ (YS) (**Figure 5B**). Soil TN content revealed that mixed cropping was significantly higher than oat monocropping and the corresponding bean monocropping in all ecological zones, but the differences among the three mixed cropping patterns were not significant. In addition, TN content increased the most in MY compared to pre-sowing (**Figure 5C**). Soil ANN and NN contents in all ecological zones were in the order of bean monoculture > mixed > oat monoculture, and soil ANN and NN were significantly greater than the pre-sowing soils, except for oat monoculture (**Figures 5D, E**). Soil MNB content increased in all ecological zones and all cropping patterns compared to pre-sowing. Among them, oat monoculture was the lowest, and the mixed cropping pattern was the highest. The cropping patterns with the highest MBN in each ecological zone were HZ (YJ), GN (YC), and HZ (YS) (**Figure 5F**). MY had significantly higher SOM, TN, NN, ANN, and MBN contents than HZ or GN (**Figure 5**).

### 3.5 Effect of the cropping pattern on biological nitrogen fixation efficiency and the amount of nitrogen fixation by the legume forage in different ecological zones

**Figure 6:**
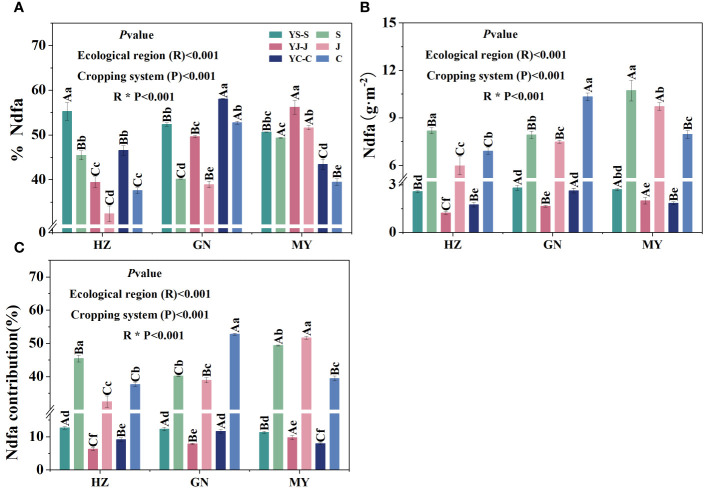
Biological nitrogen fixation characteristics of legume forage under the different cropping patterns in different ecological zones. **(A)** Biological nitrogen fixation rate. **(B)** Biological nitrogen fixation. **(C)** Biological nitrogen fixation contribution.

The cropping pattern, ecological zone, and their interactions had extremely significant (*p*< 0.01) effects on the rate of BNF, the amount of BNF, and the contribution of nitrogen fixation of legume forages (**Figure 6**).

Nitrogen fixation efficiency of legumes was significantly higher in mixed cropping than in the corresponding monocropping in all ecological zones and was significantly different among the three legumes in the same area. Among them, the cropping patterns with the highest nitrogen fixation rates in all ecological zones were HZ (YS), GN (YC), and MY (YJ), respectively. In addition, the nitrogen fixation rate of common vetch was significantly different among the three ecological zones (**Figure 6A**). BNF and the contributions of BNF were higher in all ecological intervals for monocultures than the corresponding mixtures and differed significantly among the three legumes in the same region. All three legumes in MY had higher biological nitrogen fixation rates than those in HZ and GN (except GN, fava bean). In addition, YS was the cropping pattern with the highest amount of BNF and contributions of BNF from mixed cropping in all regions. The amount of BNF and contribution of nitrogen fixation by fava bean were significantly higher in GN than in other regions (**Figures 6B, C**).

### 3.6 Effects of mixed sowing in the different ecological zones on the proportion of nitrogen transfer and the amount of nitrogen transferred by leguminous forage grasses

**Figure 7:**
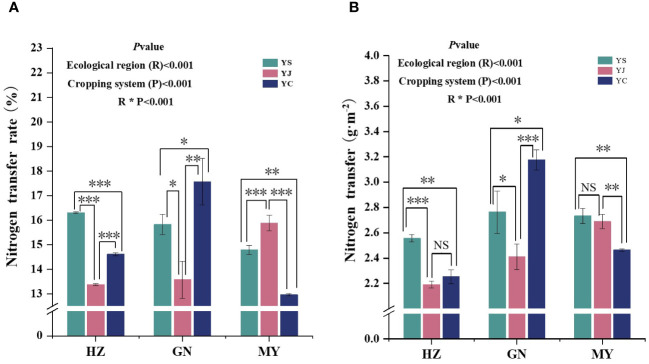
Nitrogen transfer from legume forage to oats under different cropping patterns in different ecological zones. **(A)** Nitrogen transfer rate. **(B)** Nitrogen transfer. ∗ denotes 0.01< *p*< 0.05, ∗∗ denotes *p*< 0.01, ∗∗∗ denotes *p*< 0.001, and ns denotes *p*> 0.05.

The cropping pattern, ecological zone, and their interactions had extremely significant (*p<* 0.01) effects on the N-transfer rate and the amount of N-transferred by legume forages (**Figure 7**). The nitrogen transfer rates of all three legume species were significantly different in the same region, and the order of the nitrogen transfer rates in the regions was HZ (YS > YC > YJ), MY (YC > YS > YJ), and GN (YC > YS > YJ), respectively. In addition, YS, YJ, and YC had the highest nitrogen fixation rates among the regions HZ, GN, and MY, respectively (**Figure 7A**). Significant differences in N transfer were detected among the three legume species in the same region, except for YC and YJ (HZ), and YS and YJ (MY), which were not significantly different. The order of the nitrogen transfer rate among regions was HZ (YS > YC > YJ), MY (YC > YS > YJ), and GN (YS > YJ > YC), respectively. Moreover, the highest nitrogen fixation rates were found in the regions of YS, YJ, and YC: GN, MY, and GN, respectively (**Figure 7B**).

### 3.7 Factors affecting biological nitrogen fixation and nitrogen transfer

**Figure 8:**
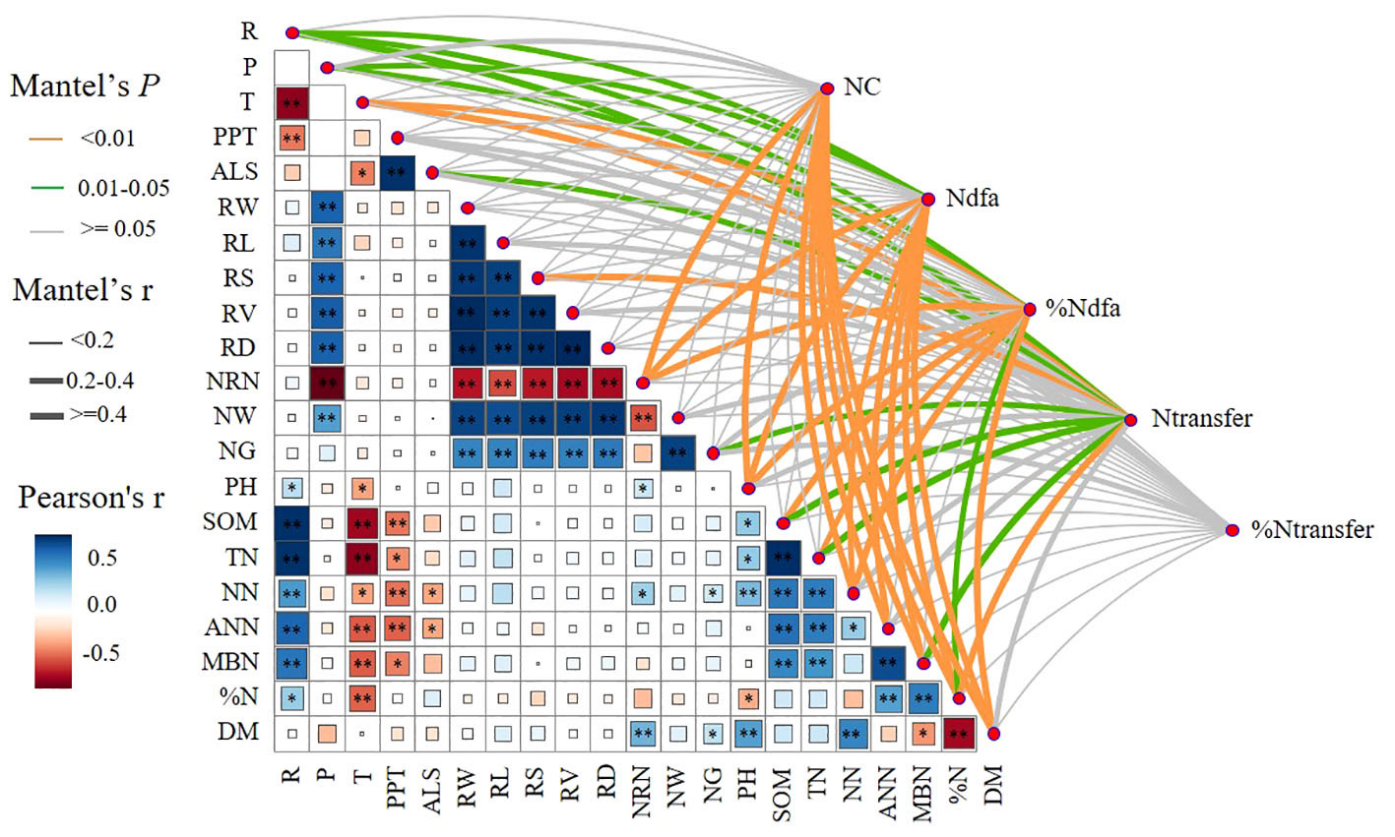
Correlation between biological nitrogen fixation, nitrogen transfer, and biological nitrogen fixation contribution with cropping system (P), ecological region (R), climatic factors, root tumor characteristics, soil physical properties, and aboveground dry biomass. T, temperature; ASL, altitude; RW, root dry weight; RL, root length; RS, root surface area; RV, heel volume; RD, root diameter; NRN, number of rhizomes; NW, fresh weight of rhizomes; NG, nitrogen-fixing enzyme activity; DM, aboveground dry biomass. The width of the Mantel edge corresponds to the Mantel r value, and the color of the edge indicates statistical significance. ∗ denotes 0.01< *p*< 0.05, ∗∗ denotes *p*< 0.01.

Pearson’s correlation analysis showed that the ecological region (R) was significantly and positively correlated (*p*< 0.05) with N%, MBN, ANN, NN, TN, SOM, NN, pH, PPT, and T. The planting pattern (P) was significantly and positively correlated (*p*< 0.05) with NW, NRN, RD, RV, RS, RL, and RW. Mantel’s test showed that NRN, pH, NN, ANN, MBN, N%, and DM were significantly and positively correlated (*p*< 0.05) with BNF contribution (NC). P, R, NRN, pH, MBN, NN, ANN, N%, and DM were significantly and positively correlated (*p*< 0.05) with the amount of nitrogen fixation (Ndfa). P, R, T, NRN, NRN, pH, SOM, TN, NN, N%, and DM were significantly and positively correlated (*p*< 0.05) with BNF (%Ndfa). R, T, ALS, RS, GN, SOM, TN, MBN, and N% were significantly and positively correlated (*p*< 0.05) with nitrogen transfer (Ntransfer) (**Figure 8**).

**Figure 9:**
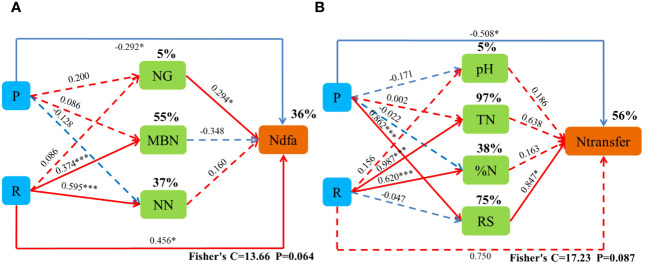
Piecewise structural equation modeling (SEM) describing the effects of the cropping system in different ecoregions on biological nitrogen fixation and nitrogen transfer in legume forage. Effect of cropping pattern (R) and ecological region (P), nitrogen-fixing enzyme activity (NG), microbial mass nitrogen (MBN), nitrate nitrogen (NN), pH (Ph), total nitrogen (TN), nitrogen content (%N), and root surface area (RS) on biological nitrogen fixation (Ndfa) and nitrogen transfer (Ntransfer). Solid lines indicate significant effects, and dashed lines indicate non-significant effects. * and *** indicate significant differences at 0.05 and 0.001 levels, respectively.

R and NG had a significant direct positive effect on Ndfa, and P had a significant direct negative effect on Ndfa. The cropping pattern indirectly affected Ndfa by increasing NG, and the ecological zone indirectly increased Ntransfer by significantly increasing NN. P had a significant direct negative effect on Ntransfer, and NW had a significant direct positive effect on Ntransfer. R increased NT by increasing N%, and P increased Ntransfer by increasing RS (**Figure 9**).

## 4 Discussion

The present study showed that the cropping pattern in different ecological zones increased crop aboveground dry matter and changed the root conformation. Mixed cropping improved soil nutrients and had beneficial effects on the rhizomes, nitrogen fixation, and nitrogen transfer. Studying the changes in nodulation, nitrogen-fixing enzyme activities, root phenotypic traits, and soil physicochemical traits in legumes helped to understand the overall response of BNF and nitrogen transfer in legumes in different ecological zones. The ability of legumes to biologically fix nitrogen is a result of their symbiosis with rhizobacteria, which are present in the rhizomes of legumes, and different crop types and growing environments affect the formation and activity of nodules, which, in turn, affects the amount of nitrogen biologically fixed by the plant (Suter et al., 2015; Duchene et al., 2017). Nitrogen fixation in the aboveground portion of legumes in an intermixed cropping system yields 40–100 kg ha^−2^, and legume nitrogen fixation in an intermixed cropping system is more than three times that in a monoculture (Jensen, 1996), whereas 7%–42% of the nitrogen in non-leguminous forage is transferred by legumes (Schipanski and Drinkwater, 2012; Thilakarathna et al., 2016). This is due to nitrogen fixation by leguminous crops, which alters the soil’s carbon-to-nitrogen ratio, improves the soil’s nitrogen balance, and accelerates the cycling of nutrients and enzymes required for normal plant growth and development, which has a positive effect on crop growth (Spiegel et al., 2007). Therefore, rational farming practices and cropping patterns not only add additional nitrogen to the soil and improve soil health but also effectively promote the agroecosystem cycle (Crème et al., 2015; Zhao et al., 2015).

Mixed cropping in all ecological regions had more aboveground dry matter than monocultures and LERs > 1, suggesting that interspecific complementarity is greater than interspecific competition in hybrid systems. Previous studies have reached the same conclusion (Willey, 1979) because plants use interspecific complementarity and interspecific competition to access soil resources and promote rhizomatous nitrogen fixation through positive plant–soil–microbe interactions (Duchene et al., 2017). Plant facilitation and competition coexist in intercropping/mixed cropping, and facilitation occurs when plant species positively interact to provide complementary services (Bedoussac and Justes, 2010; Amossé et al., 2013). The interspecific relationships of crops are related to root conformation and root depth, as root characteristics determine the depth of water and nutrient uptake (Peoples et al., 2004; Hauggaard-Nielsen et al., 2008). Plants use the plasticity of roots to avoid excessive root competition and to explore different regions of the soil, which in turn acts on the growth and development of the aboveground parts (Schroth, 1998; Hauggaard-Nielsen et al., 2001; Rich and Michelle, 2013). Our study confirmed that mixing oats with the three legumes promoted root growth, particularly increasing root length and surface area in the mixed cropping system, increasing nutrient uptake from the soil, and promoting aboveground growth. The amount of aboveground dry matter was significantly higher in all of the mixes than in monocropping and that the mixed cropping system was in an interspecific complementary situation. The aboveground N contents of oats planted under mixed cropping in the same ecoregion were all significantly higher than those of single-cropped oats, while the opposite was true for pulses, and there were differences in the N contents of oats among the three mixed cropping modes of pulses, YS, YJ, and YC. One was that N fixed by legume crops is taken up and utilized by grass crops, depleting the nitrogen in the soil and forcing legumes to increase their BNF rate to meet their own nitrogen needs, while utilization of nitrogen resources by oats is enhanced by interspecific intercropping (Wahbi et al., 2016; Ingraffia et al., 2019). Second, the N content of legumes is inherently higher than that of oats, and the difference in N between plants after mixed cropping establishes a reservoir–source relationship for oat–mungbean intercropping, resulting in the transfer of nitrogen from high- to low-N plants and an increase in the N content of the oats (Neumann et al., 2009; Rui et al., 2022).

The root tumor is the main site of BNF in leguminous plants (Gibson, 1977), and the strength of the nitrogen fixation capacity of the root tumor is related to the number of root tumors, the weight of the root tumors, the activity of nitrogen-fixing enzymes, the habitat of the plant, and the characteristics and growth and reproduction of the plant (Vincent et al., 1980), such as farming practices, cropping pattern, and climatic conditions (Wheatley et al., 1995; Marcarelli and Wurtsbaugh, 2010; Ben-Chuan et al., 2022). In this study, the number of nodules, weight, and nitrogen-fixing enzyme activity of the legumes increased in the mixed cropping system, but the number of rhizomes varied considerably from crop to crop, with pea having the highest number of nodules and fava beans the largest rhizome weight, and the differences in rhizome characteristics among the different crop species were greater than those among the different cropping patterns. This finding suggests that plant nodulation is mainly determined by genetic characteristics (Hardarson and Atkins, 2003; Keneni et al., 2013), but cropping systems can also significantly increase the number and weight of rhizomes (Fujita et al., 1992; Bloem et al., 2009). In addition, the characteristics of crop rhizomes of the same cropping pattern varied considerably in different ecological zones; particularly, the number and weight of the legume nodules were significantly higher in MY than in HZ and GN, except for fava bean, which had the highest number and weight of nodules in GN. This may have occurred because of the fertile soil and suitable climate in MY, which was favorable for legume crop nodulation, and similar results were reported by related studies (Dollete et al., 2023). Beuselinck et al. (2005) confirmed that plants grown in fertile soils have dense and large rhizomes, whereas those grown in poor soils have fewer rhizomes. However, the fava bean tumor pattern is the opposite, with the lowest temperature and rainfall in the GN region inhibiting the growth and development of the crop, and the aboveground dry matter yield of the crop in the GN (highest altitude) region was lower than in all other regions. This may be due to the fact that broad beans are more adaptable to their environment and that low temperatures and dry conditions are more favorable for nodulation. This is contrary to the findings of Lumactud et al (Fernández-Luque et al., 2008; Belén et al., 2015). This may have occurred because of the different crop species studied; broad bean has a longer root system, greater volume and surface area, greater ability to draw water and nutrients, greater ability to cope with drought, and a lower effect on nodulation (Rowse and Goodman, 1981; Nyalemegbe and Kenneth, 1994). In addition, the mixed cropping increased nitrogen-fixing enzyme activity in the rhizomes of the leguminous crops, which was consistent across all three leguminous crops in all ecological zones. Root interactions in the mixed cropping system may be responsible for the increased oat root growth, root surface area, and spatial expansion of the oat root system (Hoad et al., 2001; Gao et al., 2010), which created a favorable microaerobic environment and promoted nitrogen-fixing enzyme activity (Serraj, 2003; González et al., 2015).

Nutrient composition of the soil and physicochemical factors, such as soil organic matter (SOM), soil TN, soil total phosphorus (TP), and electrical conductivity (EC) are soil factors that significantly affect the rhizomes of legumes. In turn, the tumor characteristics of the plant counteract soil quality (David and Khan, 2001; Massawel et al., 2016). Our study found that mixed cropping significantly increased SOM, TN, nitrate–nitrogen, and ammonium–nitrogen compared to oat monoculture, but nitrate–nitrogen and ammonium–nitrogen contents were lower than those in monoculture legumes. This may have occurred because nutrients are released from the decomposition of withered material and root rot of leguminous crops, while the nitrogen fixation of legumes increases the number of beneficial microorganisms in the soil, accelerates nutrient mineralization (Wardle et al., 2006; Deyn et al., 2011; Zhao, 2014), and increases the organic matter content in the soil, providing a favorable environment for crop growth (Fontaine et al., 2003; Blagodatskaya and Kuzyakov, 2008; Bernard et al., 2009). Second, oat and legume root interactions were enhanced in the mixed cropping system and became closer as the reproductive period progressed, with root secretions inducing interactions between roots, soil microorganisms, and the surrounding soil particles (Burns, 1982; Dennis et al., 2010). Plant-secretion-enriched soil microorganisms play a key role in the decomposition of SOM and nutrient cycling by releasing and influencing various enzyme activities (Trasar-Cepeda et al., 2008; Kabiri et al., 2016; Zhou et al., 2016). Third, N released by legumes undergoes three pathways, including plant resorption–denitrification and loss-soil microbial fixation (Nasholm et al., 2009; Cameron et al., 2013). The main form of N in legume rhizome sediments is ammonium-N, which is converted to plant-available nitrogen by nitrification (Lesuffleur et al., 2007; Paynel et al., 2008), resulting in increased levels of nitrate and ammonium-N in mixed cropping systems.

The BNF rates of the three legumes varied significantly in the different ecological zones, and the BNF rates of the three legumes also varied significantly within the same ecological zone. The cultivation patterns with the highest BNF rates in each region were HZ (YS), GN (YC), and MY (YJ), respectively. This finding indicates that different crops have different adaptability to the environment and that climatic and soil factors affect nitrogen fixation in legume crops (Sprent, 1999). A rational cropping pattern will increase the nitrogen fixation rate of the crop (Zang et al., 2015; Tsialtas et al., 2018). Smercina et al. (2019) showed that BNF is cumulatively sensitive to environmental changes that affect the growth and development of the aboveground parts and the belowground root systems of crops (Smercina et al., 2019; Chun et al., 2021). Intercropping improves N fixation (%Ndfa) in legumes because legumes take up less N from the soil in intercropping systems, which improves N fixation efficiency (Danso et al., 1987). This effect becomes more pronounced as grain density increases (Fan et al., 2018). This study showed that the YS cropping pattern had the highest BNF and BNF contribution in the three ecological zones, and in the mixed cropping system with oats, the forage pea was at a disadvantage in the utilization of soil nitrogen resources, stimulating its BNF and enabling oats to obtain more ground nitrogen (Ingraffia et al., 2019). The N transfer rate and N transfer of the three leguminous crops differed significantly in the same ecological zone and also among the ecological zones of the same cropping pattern. Of these, 11.38%–12.68%, 6.85%–9.77%, and 7.94%–11.65% of the nitrogen in oats from the mixed cropping systems of YS, YJ, and YC came from S, J, and C, respectively. This is due to differences in the characteristics of the different types of legume nodules and root systems (Rao and Ito, 1998), changes in cropping patterns that can affect nitrogen fixation, and the different ecological conditions that affect root secretions. In addition, there are differences in soil nutrient cycling, the growth of aboveground parts of plants, and the uptake and translocation of nitrogen from the crop (Gupta et al., 2006; Collino et al., 2015). A high N fixation rate did not represent a high N transfer rate because N fixation is mainly associated with nodulation in legumes (Weaver, 1987), whereas N transfer is related to N uptake, the difference in N content between oats and legumes, and the closeness of root contact between the two (Stern, 1993; Shao et al., 2021).

Factors affecting nitrogen fixation and the nitrogen transfer rate in legumes are complex, with the cropping environment, cropping pattern, and crop type being the key factors (Rui et al., 2022). This study showed that the cropping pattern and ecological zone promoted BNF in legumes by increasing nitrogen-fixing enzyme activity and ammonium–nitrogen content. In addition, the ecological zone and cropping pattern improved the nitrogen transfer rate in the legumes by increasing rhizome weight and aboveground nitrogen content. In this study, we found that Legume-grass mixtures can improve the biological nitrogen fixation capacity of legume crops. By promoting rhizoma formation and optimizing root morphology, it promotes crop growth and development, increases soil nutrients and optimizes resource allocation. We also found that Legume-grass mixtures also affects root secretions, rhizobia, and nitrogen-fixing bacteria, thereby altering the inter-root microcosm. However, the molecular biological mechanisms by which nitrogen-fixing microorganisms affect legume-grass mixtures are not yet clear and can be further elucidated with the help of new technologies such as soil macrogenomics and macrotranscript genomics.

## 5 Conclusion

Legumes reduce atmospheric nitrogen to plant-available nitrogen (ammonia) catalyzed by nitrogen-fixing enzymes. Plants use interspecific complementarity and interspecific competition to access soil resources and promote the process of rhizomatous nitrogen fixation through plant-root-soil interactions. This finding provides further evidence that mixing legumes with oats is an effective sustainable forage management practice. We found significant differences in BNF rates of legumes grown with different types of legumes and in different ecological zones, but overall, mixed cropping significantly increased the BNF rates of legumes. In addition, high rates of BNF did not represent high rates of nitrogen transfer. Our study demonstrated the effects of the ecological zone and cropping pattern on BNF and nitrogen transfer in alpine mixed-seeded grassland. These results will help us to better understand nitrogen fixation and grass interactions in alpine grassland ecosystems.

## Data availability statement

The original contributions presented in the study are included in the article/supplementary material. Further inquiries can be directed to the corresponding author.

## Author contributions

FL: Data curation, Software, Writing – original draft, Writing – review & editing. WM: Investigation, Methodology, Writing – review & editing. WL: Funding acquisition, Methodology, Writing – review & editing.
